# MicroRNA: A Linking between Astrocyte Dysfunction, Mild Cognitive Impairment, and Neurodegenerative Diseases

**DOI:** 10.3390/life12091439

**Published:** 2022-09-16

**Authors:** Angelica E. Ramírez, Natalia Gil-Jaramillo, María Alejandra Tapias, Yeimy González-Giraldo, Andrés Pinzón, Pedro J. Puentes-Rozo, Andrés Felipe Aristizábal-Pachón, Janneth González

**Affiliations:** 1 Departamento de Nutrición y Bioquímica, Facultad de Ciencias, Pontificia Universidad Javeriana, Bogotá 110231, Colombia; 2 Laboratorio de Bioinformática y Biología de Sistemas, Universidad Nacional de Colombia, Bogotá 111321, Colombia; 3 Grupo de Neurociencias del Caribe, Unidad de Neurociencias Cognitivas, Universidad Simón Bolívar, Barranquilla 080002, Colombia; 4 Grupo de Neurociencias del Caribe, Universidad del Atlántico, Barranquilla 080007, Colombia

**Keywords:** miRNAs, astrocyte, lipotoxicity, mild cognitive impairment, epigenetics, biomarker, neurodegeneration

## Abstract

**Simple Summary:**

Neurodegenerative diseases are complex neurological disorders with a high incidence worldwide in older people, increasing hospital visits and requiring expensive treatments. As a precursor phase of neurodegenerative diseases, cognitive impairment needs to be studied to understand the factors that influence its development and improve patients’ quality of life. The present review compiles possible factors and biomarkers for diagnosing mild cognitive impairment based on the most recent studies involving miRNAs. These molecules can direct the gene expression in multiple cells, affecting their behavior under certain conditions, such as stressing factors. This review encourages further research into biomarkers that identify cognitive impairment in cellular models such as astrocytes, which are brain cells capable of maintaining the optimal conditions for the central nervous system functioning.

**Abstract:**

The importance of miRNAs in cellular processes and their dysregulation has taken significant importance in understanding different pathologies. Due to the constant increase in the prevalence of neurodegenerative diseases (ND) worldwide and their economic impact, mild cognitive impairment (MCI), considered a prodromal phase, is a logical starting point to study this public health problem. Multiple studies have established the importance of miRNAs in MCI, including astrocyte regulation during stressful conditions. Additionally, the protection mechanisms exerted by astrocytes against some damage in the central nervous system (CNS) lead to astrocytic reactivation, in which a differential expression of miRNAs has been shown. Nevertheless, excessive reactivation can cause neurodegeneration, and a clear pattern defining the equilibrium point between a neuroprotective or detrimental astrocytic phenotype is unknown. Therefore, the miRNA expression has gained significant attention to understand the maintenance of brain balance and improve the diagnosis and treatment at earlier stages in the ND. Here, we provide a comprehensive review of the emerging role of miRNAs in cellular processes that contribute to the loss of cognitive function, including lipotoxicity, which can induce chronic inflammation, also considering the fundamental role of astrocytes in brain homeostasis.

## 1. Introduction

Patients with mild cognitive impairment (MCI) present a cognitive decline at a greater level than expected by age without reaching dementia criteria [1]. MCI has been considered a risk factor for developing dementias, including neurodegenerative diseases (ND), such as Alzheimer’s (AD) and Parkinson’s (PD) diseases [2,3]. For example, AD has been recognized as a pathology that progresses for decades, initially manifesting as MCI [4]. However, epidemiological investigations report that 25 to 50% of PD patients presented with MCI, depending on the population and clinical setting [2,5]. In the case of all dementias, MCI is considered a prodromal stage due to the time it takes for this disease to present the first symptoms. It has been reported that the progression from MCI to dementia increases between 80 and 90% after six years [1]. About 50 million people have dementia worldwide, and it is estimated that there will be 102 million more by 2050. In addition, their treatment costs about USD 1 trillion annually [6].

Although the diagnosis of ND is carried out by clinical criteria based on the *Diagnostic and Statistical Manual of Mental Disorders (DSM-5)*, in which a significant cognitive decline affecting independence in everyday activities is highlighted, this sign is observed when the disease is already advanced. In contrast, in MCI, there is a modest cognitive decline without affecting everyday activities [7]. However, the diagnosis of MCI is performed through neuropsychological assessment tools such as the Mini-Mental Status Examination (MMSE) or the Montreal Cognitive Assessment (MoCA) [8,9,10]. Unfortunately, the first one does not have recommended specificity and sensitivity, while the outcome of the second one depends on educational level, cultural norms, and other aspects. Moreover, neuroimaging AD biomarkers and proteins in cerebrospinal fluid have been used but have not been validated in the context of MCI [4]. Therefore, identifying new biomarkers becomes imperative to improve MCI diagnosis and prognosis, preventing other ND.

Following the above, it is crucial to overcome the reductionist paradigm approach to the study of MCI, considering the participation of multiple brain cells in its development; for example, the astrocytes. These glial cells maintain the equilibrium of brain physiological characteristics [11]. Nevertheless, factors such as cellular senescence could induce function loss, causing neurodegeneration. During injury or inflammation, reactive astrocytes may cause a homeostatic disturbance [12]. Interestingly, it has been found that astrocytes play an important role in cognitive functions [13].

Due to the fundamental role performed by the astrocytes, the molecules that regulate the activation of the astrocytic metabolic pathways and pro- or anti-inflammatory mechanisms could be potential biomarkers for ND. Among these molecules are the miRNAs expressed by astrocytes, which might be suitable candidate biomarkers because they are stable in blood and cerebrospinal fluid, making them easily detected [14]. In addition, these non-coding RNAs represent an epigenetic mechanism that leads to gene silencing, affecting protein levels without altering the DNA sequence [15].

The lipidome is the set of lipids necessary for cell metabolism [16]. However, an excessive increase in lipids leads to diseases due to lipotoxicity. This toxicity of lipids is characterized by metabolic dysfunction generating mitochondrial and endoplasmic reticulum stress, autophagy, and inflammation [17]. It is important to highlight that inflammation is a particular feature of neurodegeneration [18]. Additionally, inflammation can affect astrocytes and thus lose their functionality [19]. Hence, lipotoxicity could play a key role in understanding ND by analyzing the presence of high fatty acids in astrocytes and other cells. Furthermore, the central nervous system (CNS) is significantly affected by the lipotoxic effects of dyslipidemia, which develops MCI and AD [20]. For example, it has been shown that when mice are exposed to a high concentration of lipids, only one day is needed to lose performance in memory tasks [20].

Remarkably, lipotoxicity triggered by high concentrations of saturated lipids such as palmitic acid (PA) has been related to the progression of neurodegeneration in diseases such as AD and PD [21]. It has also been associated with cognitive decline and generation of pro-inflammatory responses, causing loss of astrocytic function, where the miRNAs are possible mechanisms involved [22]. It should be noted that the expression of miRNAs in mice can be modified by diets rich in fatty acids [23]. Thus, excess lipids in the brain could be considered a starting point for the dysregulation of miRNAs and genes involved in different cellular processes.

Accordingly, a better understanding of the role of miRNAs in the development of ND will allow us to improve our knowledge of these diseases, thus enabling their diagnosis and prevention. The following sections will focus on the biological role that miRNA dysregulation exerts in the CNS, their importance in stressing conditions such as lipotoxicity, and their possible role as biomarkers in ND progression. Additionally, astrocytes will be highlighted as fundamental cells in these miRNAs’ expression and influence.

## 2. Non-Coding RNAs as Key Epigenetic Factors

Classically, epigenetics is defined as the mechanisms by which the genotype produces the phenotype, and its study is mainly centered on transcription factor activation, DNA methylation, and histone modifications [24,25]. Nevertheless, cell expression control is more complex than that, and factors such as non-coding RNAs also play an important role in the process [26]. For example, long non-coding RNA BACE1-AS is strongly related to AD, and studies have proposed it as a potential biomarker [27]. BACE1-AS can transcriptionally silence miR-214-3p, promoting the expression of ATG5 and inducing neuronal damage through Aβ_1-42_ production [28]. In addition, the expression of BACE1-AS increases the β-secretase 1 (BACE1) levels by sequestrating BACE1-targeting miRNAs [29,30]. BACE1 is reported as the protein responsible for amyloid plaque formation, and its relationship with AD pathogenesis has been documented [31]. Moreover, miRNAs are also crucial elements in controlling gene expression at the transcriptional level. For instance, miR-1, miR-22p, miR-26b-3p and miR-28-3p have a strong relationship with ND, including AD, PD, Huntington’s (HD), and amyotrophic lateral sclerosis (ALS) [32].

### miRNAs: Biogenesis and Functions

Back in the human genome project time, only 1.9% of the human DNA had a defined or predicted function [33]. Currently, we are discovering new miRNAs controlling hundreds of metabolic events daily, slowly improving our knowledge about the human genome. Nevertheless, we are far from completely knowing the direct relationship between a miRNA and a developmental process or disease [34].

The miRNA biogenesis itself is a clue to understanding how miRNAs can exert control over processes such as neurogenesis and neurodegeneration. First, RNA polymerase II or III synthesizes the primary miRNA (pri-miRNA), a hairpin loop RNA capped at the 5′ end and polyadenylated at the 3′ end. Next, pri-miRNA must be excised by the nuclear endoribonuclease Drosha in complex with the dsRNA-binding protein DGCR8, releasing an ~70 nt stem-loop precursor miRNA (pre-miRNA) with a 30 overhang. Then, the Exportin-5 and Ran-GTP complex translocates the pre-miRNA to the cytoplasm, where it is processed by the endoribonuclease Dicer, yielding an ~22 nt RNA duplex. After that, an Argonaute protein conserves the guide strand (complementary to de target mRNA), and the passenger strand is released and degraded [35,36]. Finally, the guide strand directs the mRNA degradation by Argonaute proteins through complementarity to the transcript’s untranslated (UTR) or even coding sequence (CDS) regions [37,38,39]. Each point in this process represents a regulation possibility, where even the final size of the miRNAs can affect their target specificity and, therefore, change the way a gene is expressed [40]. Hence, the epigenetic function of miRNAs has been demonstrated to be complex, and its understanding could reveal multiple critical points in some diseases.

For instance, cell differentiation can be directed through determined miRNA expression [41]. That is the case of pluripotency reprogramming using the miR-290 cluster, let-7, and miR-130/miR-301/miR-721 [42,43,44], and neuronal reprogramming into different phenotypes through let-7, miR-9-5p/3p, miR-124, and miR-218 [45]. Additionally, miRNAs can mediate the differentiation, expansion, inflammation, and apoptosis of the CNS cells, which could promote or inhibit MCI and ND [46,47].

Recently, the miRNA expression has attracted the attention of researchers in the ND field, finding some direct correlations between their presence/absence or augmentation/diminution throughout the disease progression [32,48].

## 3. The Crosstalk between MCI Factors and miRNA Dysregulation

As established before, miRNA expression responds to environmental and internal factors, which allows us to affirm that miRNA dysregulation is a multifactorial process. For example, miRNA dysregulation can be a consequence of determinate events disrupting cerebral homeostasis, including blood-brain barrier (BBB) damage, neuroinflammation, and reduced blood flow to the brain, among others. However, miRNAs can also be the cause of those events, representing a complex control loop that we need to understand to elucidate the association between MCI and ND development [49,50,51].

In the BBB context, an erythrocyte-derived miRNA, miR-451a, can reach the CNS when this barrier is damaged [52]. Interestingly, miR-451a promotes neuronal differentiation by inhibiting cellular proliferation [53], and its entrance to the CNS could lead to a differentiation imbalance. Furthermore, the cardiac overexpression of miR-1, a muscle-enriched miRNA, attenuates the synaptic vesicle exocytosis. The diminished synaptic activity could explain the relationship between cognitive deterioration and cardiac diseases, called “cardiogenic dementia” [54]. Another miRNA that alters synaptic activity is miR-128, which regulates the expression of two synaptic transmission proteins, SNAP-25 and synaptotagmin 1 (Syt1). However, an interesting experiment with mice demonstrated that miR-128 effects could be reversed by exposing the individuals to an enriched environment that offers the opportunity to express the full range of species-typical behavioral patterns [55].

Chronic low-grade inflammation is associated with increased odds of developing MCI [56]. The expression of some miRNAs has been related to an inflammatory scenario in the brain. For example, miR-31-5p is responsible for repressing the Numb expression, a Notch pathway negative regulator, degrading the Notch intracellular domain [57]. Interestingly, Notch defines whether a cell acquires neural potential during neurogenesis and if the progeny will display neural or glial fates [58]. Furthermore, the downregulation of miR-31-5p can inhibit the M1 phenotype in microglia and neuroinflammation [57]. Moreover, the attenuation of miR-31-5p expression via CircARF3 improved BBB integrity and decreased neuronal apoptosis and microglial activation in a subarachnoid hemorrhage rat model by inactivating the MyD88-NF-κB pathway [59]. Additionally, miR-15a/16-1 is associated with vascular cognitive impairment and dementia, and its genetic deletion improves the related cognitive and sensorimotor deficits of this disease. Noteworthy, miR-15a/16-1 inhibits AKT3 and IL-10RA expression, known anti-inflammatory proteins [60].

Moreover, opioid abuse can trigger neuroinflammation, making it a possible cause of MCI. That is the case of morphine-mediated microglial activation, where miR-138 is released by astrocytes, activating the TLR7 receptor in BV-2 microglial cells. These findings were confirmed in vivo by administrating morphine in wild-type mice, resulting in increased microglial activation in the thalamus, which has been related to MCI and dementia development in humans [61,62,63]. Interestingly, miR-124 and its target, IQGAP1, have shown a regulative role in addition and cognitive impairment in morphine-dependent patients [64]. Furthermore, ethanol drinking also causes neuroinflammation and brain damage by TLR4 activation. Murine astrocyte treatment with ethanol increased miR-146a, miR-182, and miR-200b levels in extracellular vesicles. This change increased the expression of the inflammatory-related proteins TLR4, NFκB-p65, IL-1R, caspase-1, and NLRP3, reducing neuronal survival [65].

Ischemia is a mechanism derived from impaired blood flow to the brain, causing an acute brain injury. The lack of oxygen and glucose supply induces the loss of neurons and gliosis, and the extent of neuronal damage depends on the area size and duration of the hypoperfusion [66]. In this sense, an ischemic lesion can increase the risk of developing MCI and, after that, some dementia [67]. For that reason, tracking miRNA dysregulation in ischemic injuries can shed light on the process of developing MCI. For instance, miR-143-3p is aberrantly expressed in patients with ischemic stroke, playing an essential role in angiogenesis [68,69]. Its reduction via the circular RNA circ_0025984 protects astrocytes from ischemia-induced autophagy and apoptosis [69]. Furthermore, miR-125a-5p, miR-125b-5p, and miR-143-3p were found to be upregulated in ischemic patients and patients with transient ischemic attacks. In an interesting way, miR-125b-5p and miR-143-3p return to control levels two days after the lesion, making them good candidates for ischemic biomarkers [70].

According to this, astrocytic miRNA expression has a fundamental role in the ND progression and in processes that trigger MCI, such as BBB disruption, ischemia, and inflammation.

## 4. Structure and Function of Astrocytes

Astrocytes, also known as astroglia or astroglial cells, are the most abundant type of glial cell in the brain [71], being diverse in their ability to regulate and support several functions in the CNS [72]. For example, these cells maintain brain homeostasis and neuronal metabolism, supporting the neurons and the microarchitecture of the brain parenchyma [12]. Additionally, astrocytes regulate the concentrations of ions, neurotransmitters, and metabolites, controlling neural development, plasticity, and synaptogenesis [73,74,75]. Even more, it has been recently suggested that astrocytes play a role in the regulation of the sleep/wake cycle through Ca^2+^ signaling [76,77]. Furthermore, astrocytes also play a role in immune defense by, for example, recruiting neutrophils or neuronal protection and barrier formation during infection [78,79,80,81]. In addition, mature astrocytes also activate neurogenesis and support neuronal differentiation and maturation, which is a promising approach for repairing spinal cord injury [82,83,84].

Considering the heterogeneity of this cell population, it is not surprising that their disorders are related to a wide range of neuropathologies. For example, brain diseases are characterized by the active inflammatory state of the astrocytes, which presents a high expression of the glial fibrillary acidic protein (GFAP) [85,86], and many other molecular markers [87]. In particular, the loss of astrocyte function due to cellular senescence could have implications for neurodegenerative disorders, such as AD and HD, and the aging brain [88]. Additionally, the disease severity depends on the astrocytic Ca^2+^ signals driving the induction and the progression of the inflammatory state [12].

While their role in ND was thought to be deleterious, it is now believed to be more complex, depending on the stage of the disease [89,90]. Similarly, various other factors have demonstrated importance, such as the microenvironment and localization in which the astrocyte is found [91]. Therefore, astrocytes are rapidly becoming an exciting area considering their potential as targets in ND.

### miRNA Role in Astrocytes

As mentioned previously, astrocytes play a critical role in the homeostasis of the central nervous system. Both the regulation of their function and dysfunction can be mediated by miRNAs. For this reason, the expression of miRNAs in astrocytes is related to different pathologies such as AD and PD, among others [92]. Several studies have evaluated the expression profile of miRNAs in body fluids, but few have been conducted on astrocytes. Evaluating the miRNA expression directly in these cells could be useful to diagnose ND, assessing the biological processes that trigger these pathologies from the reactive astrocytes and promoting the finding of novel therapeutic targets. For instance, reactive gliosis is a pathological hallmark of ND, presented as a response to damage such as neurodegeneration. Those neuronal lesions, in turn, can be induced by the loss of the functions of the astroglia [93].

The expression of miRNAs in astrocytes depends on the brain regions where astrocytes are located and cerebral maturity. The fetal brain has a different miRNA profile in comparison with the adult brain. Likewise, there is a differential expression of miRNAs between white matter and gray matter [94]. Additionally, astrocytes are a suitable study object because they represent most brain cells and perform relevant functions [95].

Moreover, it has been found that the differential expression of miRNAs can contribute to the loss of cellular functions in astrocytes, triggering ND development [96]. For example, miR-125b and miR-449 are involved in structural function in astrocytes, attracting attention due to the assumption that their dysregulation would affect different functions and damage the physical structuring of the brain [94]. Furthermore, astrocytes have the ability to synthesize cholesterol and provide it to neurons [97], but in aged astrocytes, it has been observed dysregulation in cholesterol synthesis [98]. Interestingly, the miR-335 was found to be overexpressed in cultures of aged astrocytes, which has been associated with altered cholesterol metabolism in astrocytes and cognitive function [98].

Furthermore, CCL5 is associated with the regulation of glutamatergic transmission [99], which is secreted by astrocytes under pathological conditions [100]. CCL5 is controlled by miR-324-5p [101]. Therefore, it is coherent to infer that this miRNA affects the synapse processing in astrocytes. Another example is miR-137, which targets glutamate transporter in neurons [102], whose function is to regulate glutamate concentrations at the synapses [103]. As astrocytes are releasers of these transporters [104], there is a possibility that miR-137 affects the tripartite synapse. Moreover, miR-223 controls the NMDA-induced calcium flux in the neuronal hippocampus [105], which could be related to controlling glutamate amounts.

Another key molecule is GFAP, which is part of the formation, dynamics, and structure of the cytoskeleton of astrocytes, and it is highly expressed in the process of astrogliosis [106]. Moreover, in AD, the upregulation of the cytoskeleton is a feature of reactive astrocytes [107], which could, in turn, result in the deregulation of GFAP. Regarding miRNAs, miR-145 is reported as a regulator of the cellular dynamics of astrocytes, being a clue in astrogliosis [108]. Moreover, astrogliosis is characterized by vimentin and GFAP increase due to astrocyte proliferation. Interestingly, there is a significant correlation between the increase of these globular proteins and the over-expression of miRNA-125b [109].

In addition, different miRNAs have been involved in pathophysiological events during the development of ND by activating glial cells, including astrocytes. That is the case of miR-155, a promoter of pro-inflammatory cytokines through the modulation of SOCS-1 [110]. Similarly, miR-9 targets MCPIP1, contributing to the degradation of cytokines such as IL-6 and IL-1β [111]. Additionally, in a study where the astrocytes were treated with lipopolysaccharide (LPS), miR-181 was downregulated, triggering an increased release of TNF-α [112]. As these molecules contribute to the development of neuroinflammation, it suggests that miR-155, miR-9, and miR-181 would affect the immune response in cells such as astrocytes and thus influence the evolution from MCI to AD.

However, we cannot ignore the protective and homeostatic role of astroglia, which is also modulated by miRNA expression. For example, the astrocyte-derived miR-873a-5p promotes a microglial M2 phenotype after a traumatic brain injury, which means a less inflammatory and more repairer phenotype [113]. Interestingly, astrocytes can also modify the miRNA expression in response to inflammation. For instance, a study demonstrated that in the presence of IL-1β and TNF-α, astrocyte-derived extracellular vesicles contained higher concentrations of miR-125a-5p and miR-16-5p. These two miRNAs target NTKR3 and its downstream effector Bcl2, associated with neuronal growth and activity. In contrast, in the presence of ATP, the astrocyte-derived extracellular vesicles had the opposite effect. Additionally, this ATP challenge increased astrocytic intracellular levels of let-7f, miR-100, miR-23a, and miR-145. These changes adjust the activity of target neurons, controlling the transcriptional programs related to synaptic stability and neuronal excitability [114]. Note of worth, miR-16-5p has been associated with cell cycle G1/S phase arrest and apoptosis by targeting Bcl2, impairing fracture healing [115].

Some miRNAs are related to protection against the ischemia-triggered processes. For instance, miR-29a, enriched in astrocytes, protects neurons from forebrain ischemia by reducing the expression of the pro-apoptotic PUMA. Additionally, this miRNA decreased cell injury and improved mitochondrial function after ischemia-like stresses in vitro [116]. In addition, the loss of miR-29b contributes to neural cell death and infarct size after acute ischemic stroke. miR-29b is required for glutathione and 12-lipoxygenase-dependent arachidonic acid metabolism, leading to neurodegeneration [117]. Similarly, miR-17-5p showed neuronal protection from hypoxic-ischemic brain damage in neonatal rats. This miRNA inhibits BNIP2, a member of pro-apoptotic BNIP families, reducing neuronal apoptosis and inflammation [118]. In addition, miR-92b-3p is also associated with the protective function of astrocytes during oxygen and glucose deprivation through exosome release. This effect was tested using rat embryo neurons, which showed higher viability when exposed to miR-92b-3p-containing exosomes [119]. Interestingly, miR-92b-3p is downregulated after acute spinal cord injury, but its upregulation leads to PTEN inhibition and AKT phosphorylation, related to neurite growth and functional recovery [120].

It is worth mentioning that astrogliosis also has a beneficial role that is essential for functions in the brain, although it has detrimental outcomes under peculiar circumstances [121]. There are some miRNAs involved in both processes. For example, miR-21 has an elevated expression in astrogliosis when an injury in the spinal cord occurs. In contrast, in healthy conditions, this miRNA is diminished [122]. Similarly, miR-181 increases its levels under the influence of cytokine IL-10 [112], suggesting that this miRNA promotes inflammation. On the flip side, miR-190 reduces neuroinflammation, according to a murine model study [123]. Therefore, it is possible to infer that dysregulation in these and other miRNAs would result in a bigger injury or inefficient containment of some trauma.

Considering all the information above, we suggest that the regulation and dysregulation of miRNAs in astrocytes must be studied more in the development of MCI and ND.

## 5. Lipids in the Brain

Lipids are considered fundamental for the functioning of the brain [124]. For example, they have functions at the structural level in the lipid bilayer of the cell membrane and other organelles. In addition, they act as a source of energy storage in the CNS [16]. Furthermore, lipids contribute to the maintenance of physiological conditions, such as synapse and neuron formation, as well as cell signaling, which are relevant for various neurological disorders [125]. Dysregulation of lipidic composition could be triggered by different disorders, such as obesity [124]. For example, an imbalance of palmitate in human CSF has been observed in obese people with amnestic MCI; remarkably, the palmitate injection induced memory loss in mice [126].

In addition, the brain represents an important lipid reservoir together with adipose tissue [127]. Moreover, within the brain, specific cells, such as neurons and glial cells, have a connection with lipid metabolism [128], which work together as a metabolic unit by releasing/consuming lipids as an energy resource. In that context, astrocytes accumulate lipid droplets necessary for energy production and membrane synthesis [129]. Moreover, lipid droplets are also formed as a stress mechanism by astrocytes [130], protecting the CNS from oxidative stress and lipotoxicity [131].

Lipid droplets are also associated with high lipid diets since a high exogenous number of fatty acids contributes to their formation [130]. In addition to this, lipid droplets can be triggered as a cause and consequence of inflammatory processes [132]. In this way, it is related to ND, such as AD, suggesting lipid droplets are responses to lipid accumulation and neuroinflammation [133]. Therefore, it is also necessary to explore miRNA in cellular models such as glial cells and astrocytes under different experimental conditions, including lipotoxicity, to understand the cellular mechanisms that lead to the development of these pathologies.

As obesity and metabolic dysregulation are risk factors for cognitive decline [134], high lipid concentration could take part in the loss of cognition in older adults. Therefore, analyzing the behavior of miRNAs under these conditions might be a suitable alternative for the early identification of MCI. Furthermore, it has been proposed that the connection between the adipose tissue and the CNS, along with signals and cellular intermediaries, contributes to cognitive decline and neurodegeneration [135].

Adipogenesis, characterized by the formation of lipid droplets, is modulated by different miRNAs, such as miR-146a-3p, miR-4495, miR-4663, miR-6069, and miR-675-3p [136]. Interestingly, miR-146a was previously associated with cognitive impairment [137].

As a feature of cognitive decline and ND, neuroinflammation and some adipokines, such as adiponectin and leptin, play a part in cognitive functions [138,139]. Thus, higher levels of leptin have been observed in people suffering from cognitive impairment [134], whereas higher levels of adiponectin improve cognition [140,141]. Moreover, according to the KEGG database, there are diseases related to leptin in the JAK-STAT signaling pathway, among which obesity and MCI can be highlighted. According to the miRDB database, miR-146-5p modulates the LEPROT gene, which is associated with the overlapping transcription of the leptin receptor, whose function is associated with the immune response in the CNS [142]. Additionally, miR-378 is involved in adiponectin regulation [143], and this miRNA expression is deregulated in disorders such as AD, affecting neurogenesis [144].

Palmitic acid (PA), the most common fatty acid in the human body, has been reported to be increased in the CSF of obese people, leading to altered cognitive properties in mice [126]. It has been shown that, at healthy levels, PA plays an essential role in memory and learning and is usually acquired through diet [145]. In addition, PA induces the expression of α-synuclein, tyrosine hydroxylase, dopamine, and serotonin, which play a vital role in the development and reduction of ND [21]. Regulation of α-synuclein expression is given by miR-7, and the decrease of this miRNA is related to PD [146]. Additionally, miR-16 regulates serotonin transporters [147] and β-amyloid protein, which control cell death in an in vitro model of AD [148]. Moreover, insulin resistance increases the likelihood of MCI development [149], and some studies have observed miRNAs that prompt insulin resistance caused by high levels of PA, such as miR-3180-3p and miR-4632-5p [150], which could be related to cognitive impairment. In fact, miR-3180 has been reported to be downregulated in AD from the human cerebral cortex sample [151]. Additionally, in an in vitro model of astrocytes, high PA levels affect the expression of two miRNAs, miR-125a and miR-155-5p, which are associated with the regulation of inflammatory genes [152]. Interestingly, in microglial cells, high PA concentrations increased the miR-124 expression, which was associated with a reduced inflammatory process in these cells [153].

## 6. The Biomarker Potential of miRNAs in CNS

MCI is a multifactorial disease, including age, genetics, and educational level [154], and different miRNAs could be implied in the outcome of those factors. For example, differentially expressed miRNAs related to aging, including miR-146a-5p, miR-30a-3p, miR-148a-3p, miR-130b-3p, miR-181a-5p, and miR-192-5p, have been suggested as candidates for biomarkers of cognition [155].

Many miRNAs dysregulated in AD participate in distinct biological processes in the brain. For instance, miR-146a, enriched in microglia and neurons, is involved in the activation of glial cells and inflammatory processes [137,156]. Interestingly, miR-146a-5p is associated with a risk of loss of cognitive function [155]. Therefore, it would be interesting to relate specific miRNAs expressed in MCI to understand better the origin of the disease’s progression. In addition, miR-148a-3p is overexpressed under neurodegenerative conditions, altering the extracellular matrix. This condition would affect cellular functions such as cell movement and multiplication [155].

Recent studies have highlighted the miRNA impact on ND. For instance, a work identified a miRNA signature composed of 3 miRNAs (miR-181a-5p, miR-146a-5p, and miR-148a-3p), which alter cellular processes involved in cognition in experimental models [155]. In another study, miR-567 was upregulated in CSF, blood, and serum samples from MCI-AD patients. This work suggested this miRNA as an early development biomarker of AD due to its expression was higher in patients than in healthy people [157]. Additionally, the overexpression of miR-142-3p in the hippocampus or plasma from AD and MCI-AD patients was confirmed through a meta-analysis that included 18 studies with 1027 participants. This study showed the miR-142-3p biomarker potential with a sensitivity and specificity of 100% and 77%, respectively. The same data compilation also found miR-483-5p, which reached 100% sensitivity, and miR-107, with a 79% sensitivity for AD and 98% for MCI [46]. In addition to its high sensitivity, miR-483-5p showed the highest expression among the altered miRNAs in AD [158], reaffirming its potential role in preventing AD.

Research related to PD diagnosis has also been conducted, with the critical limitation of a few participants. A study involving 75 PD patients and 73 normal controls observed a reduction in miR-153 and miR-223 levels in the PD cases compared to control and PD-treated subjects [159]. The Additionally, the detection in CSF of the miRNA combination let-7f-5p, miR-27a-3p, miR-125a-5p, miR-151a-3p, and miR-423-5p showed 90% sensitivity and 80% specificity in a study with 40 PD patients and 40 matched control individuals [160].

Otherwise, miRNA dysregulation has also been detected in ND progression, even showing a differential expression in their preclinical and successive stages. For that reason, miRNAs are starting to attract interest with diagnosis and prognosis ends.

For example, miR-92a-3p, miR-181c-5p, and miR-210-3p have been upregulated in AD. Interestingly, MCI subjects that progress to AD showed higher levels of these miRNAs than before developing the disease. The study included 38 healthy donors, 56 AD, 26 MCI, and 27 frontotemporal dementia patients, proposing miR-92a-3p, miR-181c-5p, and miR-210-3p as potential biomarkers for AD [161]. Additionally, a more recent study with three different cohorts and 269 participants found a correlation between β-amyloid (Aβ) load and miR-27a-3p, miR-27b-3p, and miR-324-5p. Moreover, miR-195-5p and miR-335-5p were consistently upregulated in MCI, cognitively normal Aβ-positive, and AD individuals. More interestingly, miR-122-5p levels increased as the disease progressed, while miR-27b-3p, miR-29c-3p, miR-143-3p, and miR-324-5p decreased [162]. Remarkably, miR-195 inhibition ameliorates mitochondrial dysfunction in an AD mice model, improving cognitive function [163]. In addition, miR-384 has been proposed as an AD biomarker, which can be detected in exosomes in plasma blood in conjunction with other marker proteins. This method showed high power for detecting subjective cognitive decline, MCI, and AD [164].

Additionally, miR-9, miR-29a, miR-34a, miR-125b, miR-146a, and miR-29b, were evaluated in CSF due to their presence in the human brain and their relationship with AD processes. After assessing them through RT-qPCR, these miRNAs were proposed as landmarks in AD [165]. Moreover, miR-206 was found in blood plasma from patients with MCI, who also progressed to dementia over four years [166]. Therefore, these studies give us an idea of the importance of evaluating the expression of miRNAs in the MCI.

Nevertheless, there are also miRNAs with a neuroprotective role in AD. For example, the neuron-expressed miR-132-3p was found to be progressively downregulated as the disease evolved. miR-132-3p targets FOXO1a, Tau, EP300, and SIRT1, with the first protein being increased in late-onset AD patients [48]. Additionally, miR-132-3p-targeted FOXO3a induces apoptosis in cultured primary neurons, demonstrating a neuroprotective role of this miRNA [167]. Moreover, let-7a-5p has demonstrated a regenerative role for the neurons in the spinal cord in a rat model. let-7a-5p inhibits the expression of HMGA2, finally downregulating the TGF-β/Smad signaling pathway [168]. TGF-β is associated with the lesions after spinal cord injury and the formation of glial scars [169,170]. In addition, miR-107 directs BACE1 degradation, increasing cell survival, reducing lactate dehydrogenase leakage, inhibiting apoptosis, and reducing Aβ production in AD. In fact, miR-107 is a current study target for pharmacologic treatment development [171].

Furthermore, some miRNAs are dysregulated in ALS patients. For example, in a study with 48 ALS patients, 16 disease mimics, and 24 age- and sex-matched healthy controls, miR-16-5p, miR-21-5p, and miR-92a-3p were downregulated while miR-206 was upregulated only in the ALS patients [172].

In the case of multiple sclerosis, severity can be followed by the expression of some miRNAs. That is the case of miR-375 and miR-629-5p, which positively correlate with brain atrophy, while miR-143-3p, miR-142-5p, miR-181c-3p, and miR-181c-5p demonstrate a protective correlation [173]. Moreover, the upregulation of miR-155, miR-153, miR-361-5p, miR-4668-5p, miR-8071, miR-197-5p, miR-145, miR-181, miR-199a, miR-1183, miR-129-2-3p, and miR-143-3p and the downregulation of miR-134, miR-0067835, and miR-153 have been proposed as prognostic biomarkers of Mesial Temporal Lobe Epilepsy in potentially epileptogenic patients [174]. Noteworthy, miR-155 has been detected in the neurovascular unit of individuals with multiple sclerosis, and its upregulation is related to the loss of BBB function during neuroinflammation [175].

## 7. miRNAs Altered in MCI and ND: Related Biological Processes

**Figure 1:**
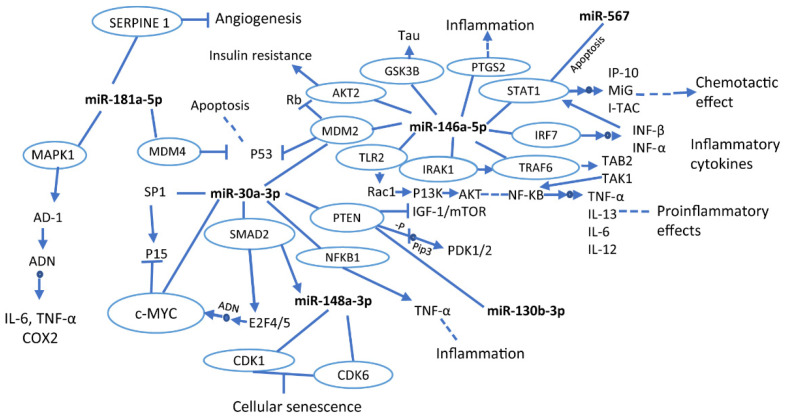
Interactions of miRNAs in signaling pathways and cellular processes. The blue circles indicate genes that are being modulated by miRNAs. The full arrows indicate production or direct activation of a particular process, while the dotted arrows show that the pathway is activated, but different processes occur prior to its termination. Likewise, the small blue circles in the middle of the arrows indicate direct action on the track. Moreover, the line terminated by another line perpendicularly means inhibition. This graphic was made based on the KEGG database.

The mentioned miRNAs are associated with different characteristic signaling pathways in the development of neurodegeneration, such as inflammation, insulin resistance, angiogenesis, and cellular senescence, through the control of several genes. Figure 1 shows a simplified summary of pathways and cellular processes of several discussed miRNAs related to neurodegenerative processes. According to the KEGG database, these miRNAs control several genes in common. That is the case of the PTEN gene, which is regulated by miR30a-30 and miR-130b-3p and involved in IGF-1/mTOR inhibition and PDK1/2activation. IGF is a gene that contributes to brain development and immune response; under normal physiological conditions, this gene is essential for brain growth and proper functioning of the central and peripheral nervous system [176].

The MDM2 gene is also regulated by miR-30a-3p and miR-146a-5p. This gene inhibits the production of p53 and Rb. The p53 is involved in the promotion of neuronal plasticity [177]. Thus, the lack of control of p53 may lead to the loss of neuronal communication, which could trigger cognitive impairment. Not having been studied yet, it could be an object of study.

The main cellular mechanisms related to these miRNAs are inflammatory processes, chemotactic effects, senescence, and cell death. Inflammatory processes are characteristic events of early AD or MCI development, and it is a crucial factor in the progression of the disease. Therefore, it has been detected as the earliest biomarker of the disease [178]. Furthermore, apoptosis is a relevant process associated with neuronal loss of neurodegeneration development [179]. In addition, cellular senescence could induce the loss of function of brain cells such as astrocytes, causing neurodegenerative disorders [12]. Finally, chemotactic activity has been reported to represent an early response to β-amyloid deposition, which suggests that miR-146a-5p in MCI could play an essential role in the early detection of AD [180].

Table 1 presents a summary of the miRNAs associated with ND; it shows their function and the diseases in which they were studied. Noteworthy, the miR-125b, miR-143-3p, miR-155, miR-181c-5p, and miR-92a-3p are related to more than two ND, which might suggest their biomarker potential in the early stages of illnesses. Inflammation, synapse reduction/augmentation, apoptosis/cell survival, and neurodegeneration are the most common functions involved with these miRNAs. All studies considered in this review were conducted using human, rat, or murine models.

## 8. Conclusions

Epigenetics plays an essential role in the regulation of different cell functions, which are affected due to gene silencing through the down/upregulation of miRNAs, among other mechanisms [202]. In addition, these miRNAs are involved in biological processes such as apoptosis, cell differentiation, and proliferation, whose dysregulation can contribute to the development of pathologies [15].

**Figure 2:**
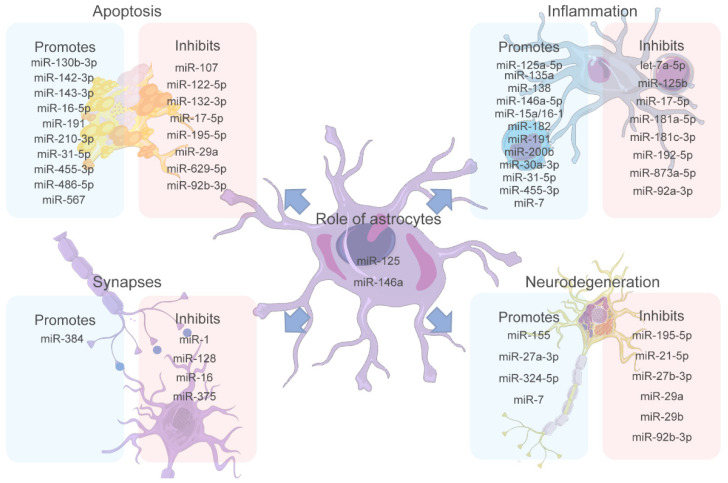
The miRNA role in significant factors triggering MCI. miRNAs play an important role in the modulation of the different physiological processes of the central nervous system (CNS) affecting different cells. Apoptosis, inflammation, synapses, and neurodegeneration are the most studied processes in MCI regarding how they are affected by miRNA dysregulation. Recent studies have revealed miRNAs that promote or inhibit each of these processes, making them potential targets for biomarkers and treatment targets. Additionally, miR-125 and miR-146a are proposed as miRNAs of great importance since they are expressed by astrocytes, essential cells for brain homeostasis, and have been shown to affect all the other processes mentioned. Figure 2 was partly generated using Servier Medical Art, provided by Servier, licensed under a Creative Commons Attribution 3.0 unported license.

Figure 2 summarizes the four main biological processes controlled by miRNAs discussed here, which are divided into those triggered and those impaired by miRNA expression. The need to study differential expression of miRNAs in specific cells such as astrocytes is evident since several miRNAs are related to the balance or imbalance in their functions, with a probable consequence in developing MCI and ND. For instance, some of the miRNAs involved in MCI have been highlighted, such as miR-146a, which is differentially expressed in glial cell activation, cognitive processes, and ND [137]. Additionally, miR-125 is reported as a structural modulator of astrocytes, and, in turn, it has been involved in MCI development [109]. Therefore, a strong hypothesis highlights these miRNAs as important study molecules in these neuronal pathologies such as MCI. Furthermore, one of the mechanisms inducing inflammation in astrocytes is related to a high concentration of lipids that alter insulin resistance. The miR-146a is also related to insulin resistance as a modulator of the AKT2 gene that controls that resistance. Moreover, from an inflammatory and immune response point of view, both miRNAs (mir-146a and miR-125b) are highly expressed. Then, substantial evidence allows us to point them out as possible markers under pathological conditions and inflammatory processes presented in the MCI, which requires further studies.

To date, differential expression of miRNAs in ND has started to be studied, but it has become pivotal to study them in MCI due to the necessity of an earlier understanding and diagnosis of these diseases. Hence, finding innovative models to improve biomarker and therapeutic target detection is essential. Many studies have been conducted under a simplistic view, focusing only on detecting the miRNAs in fluids or considering the neuron alone. Different miRNAs in MCI in human, mouse, and rat models have mostly been found in CSF and plasma samples. A more holistic view, taking into account other brain cells and their crosstalk, is required to improve the current knowledge. In this context, astrocytes are of great interest due to their fundamental role in the CNS and their involvement in the pathology of ND. Thus, these cells are considered key to the understanding of neurological disorders.

Considering the findings about miRNAs, studying and understanding the expression and interaction of these molecules in the different cellular pathways involved in MCI will allow us to understand early neurodegenerative stages. In addition, miRNAs present undeniable potential as biomarkers and could be used as pharmacological targets. Notably, multiple studies have demonstrated that a large number of miRNAs can affect biological processes and pathways, and a single miRNA can control the function of different genes that, in turn, cause physiological changes, altering homeostasis.

## Figures and Tables

**Table 1:** miRNA role in neurodegenerative diseases.

miRNA	Function	Disease	Organism	References
let-7a-5p	TGF-β/Smad signaling pathway regulation	Alzheimer	Rat	[168]
miR-1	Synaptic vesicle exocytosis attenuation	MCI-Cardiogenic dementia	Mouse	[54]
miR-107	Cell survival increase, lactate dehydrogenase leakage reduction, apoptosis inhibition	Alzheimer	Mouse	[171]
miR-122-5p	PI3K-Akt signaling pathway	MCI-Alzheimer	Human	[162,181]
miR-125a-5p	Inflammation, neuronal growth and activity	MCI-Ischemia	Human, rat	[70,114]
miR-125b	TGF-β signaling pathway	MCI-Alzheimer -Ischemia	Human	[70,137,182]
miR-128	Synaptic transmission reduction	MCI	Mouse	[55]
miR-130b-3p	Apoptosis	MCI	Human	[155]
miR-132-3p	Apoptosis inhibition	MCI-Alzheimer	Human	[48,167]
miR-135a	JAK2/STAT3 and ERK1/2 pathways	Alzheimer	Human	[137,183]
miR-138	Microglial activation	MCI	Mouse	[61]
miR-142-3p	Apoptosis	MCI-Alzheimer	Human	[46,184]
miR-143-3p	Angiogenesis, autophagy, and apoptosis increase	MCI- Alzheimer–Ischemia-Multiple sclerosis-Mesial Temporal Lobe-Epilepsy	Human, rat	[69,70,162,173,174]
miR-146a-5p	Inflammation	MCI-Alzheimer	Human, mouse	[65,137,155]
miR-148a-3p	Senescence	MCI	Human	[155]
miR-155	Neuroinflammation and neurodegeneration, BBB function loss	MCI-Alzheimer-Mesial Temporal Lobe Epilepsy-Multiple sclerosis	Human	[137,174,175,185]
miR-15a/16-1	Inflammation	Vascular cognitive impairment and dementia	Mouse	[60]
miR-16	Regulation of the serotonin transporter	Alzheimer	Rat	[148]
miR-16-5p	Inflammation, neuronal growth and activity, cell cycle arrest, and apoptosis	MCI-ALS	Rat, mouse	[114,115,172]
miR-17-5p	Neuronal apoptosis and inflammation reduction	MCI-Ischemia	Rat	[118]
miR-181a-5p	Angiogenesis, TGF-β signaling pathway	MCI	Human	[4,155]
miR-181c-3p	Axonal guidance signaling, TGF-β signaling	Multiple sclerosis	Human	[173,186]
miR-181c-5p	Neurotrophin signaling pathway	MCI-Alzheimer-Multiple sclerosis	Human	[4,161,173]
miR-182	Inflammation	MCI	Mouse	[65]
miR-191	Apoptosis, inflammation	MCI	Human, rat	[182,187]
miR-192-5p	TGF-β signaling pathway	MCI	Human	[155,188]
miR-195-5p	β-amyloid load and tau hyperphosphorylation reduction, and apoptosis inhibition	MCI-Alzheimer	Human	[162,189,190]
miR-200b	Inflammation	MCI	Mouse	[65]
miR-210-3p	Apoptosis/proliferation	MCI-Alzheimer	Human, rat	[4,161,191]
miR-21-5p	Axonal regeneration, neuronal protection	ALS	Human	[172]
miR-27a-3p	β-amyloid load increase	Alzheimer	Human	[162]
miR-27b-3p	Neurotrophin signaling pathway, β-amyloid load decrease	MCI-Alzheimer	Human	[4,162]
miR-29a	Apoptosis reduction, mitochondrial function improvement	MCI-Ischemia	Rat, mouse	[116]
miR-29b	Arachidonic acid metabolism, neurodegeneration reduction	MCI-Ischemia	Rat, mouse	[117]
miR-29c	Neurite outgrowth promotion	MCI-Alzheimer	Human, mouse	[162,192]
miR-30a-3p	Inflammation	MCI	Human	[155]
miR-31-5p	Inflammation, BBB integrity impairment, apoptosis increase	MCI	Rat, mouse	[57,59]
miR-3180	Neurotrophin signaling pathway	Alzheimer	Human	[150,151]
miR-324-5p	β-amyloid load increase	Alzheimer	Human	[162]
miR-335-5p	Key regulator of AD-related gene networks	MCI-Alzheimer	Human	[162,193]
miR-375	Aralkylamine N-acetyltransferase expression and melatonin secretion decrease	Multiple sclerosis	Human	[173,194]
miR-384	Long-term potentiation	MCI-Alzheimer	Human, rat	[164,195]
miR-451a	Neuronal differentiation promotion	MCI	Mouse	[53]
miR-455-3p	Apoptosis, inflammation	MCI	Human, rat	[4,196]
miR-4632-5p	Insulin resistance	MCI	Human	[149,150]
miR-486-5p	Apoptosis increase, aging process	ALS	Human	[197,198,199]
miR-567	Apoptosis	MCI-Alzheimer	Human	[157]
miR-629-5p	Apoptosis inhibition	Multiple sclerosis	Human	[173,200]
miR-7	Cellular ROS	Parkinson	Human, mouse	[146,201]
miR-873a-5p	Repairer phenotype	Traumatic brain injury	Human	[113]
miR-92a-3p	TGF-β signaling pathway	MCI-Alzheimer-ALS	Human	[4,161,172,199]
miR-92b-3p	Cell viability increase, neurite growth, and functional recovery	MCI-Ischemia	Rat	[119,120]

MCI: Mild cognitive impairment; BBB: Blood-brain barrier; AD: Alzheimer’s disease; ALS: Amyotrophic Lateral Sclerosis; ROS: Reactive oxygen species.

## Data Availability

Not applicable.
